# Impact of variation in the α/β of cervical cancer on predicted clinical outcomes

**DOI:** 10.1002/acm2.70608

**Published:** 2026-05-05

**Authors:** Cameron Thayer‐Freeman, Brien Washington, Dennis Cheek, Wei Luo

**Affiliations:** ^1^ Department of Radiation Medicine University of Kentucky Lexington Kentucky USA; ^2^ Department of Radiation Oncology UT Southwestern Medical Center Dallas Texas USA; ^3^ Department of Radiation Oncology University of Alabama Birmingham Alabama USA

**Keywords:** α/β, biological effective dose, cervical cancer, tumor control probability

## Abstract

**Background:**

To improve the effectiveness and efficiency in radiation therapy, various treatment modalities and fractionation schemes have been introduced and combined for cancer treatment. Biological effective dose (BED) and equivalent dose in 2 Gy fraction (EQD_2_) are used to evaluate and compare different modalities and fractionations and also used to determine dose prescriptions for new radiation schemes. BED and EQD_2_ are functions of α/β and the accuracy of α/β value is essential. A single α/β value of 10 Gy has been used for cervical cancer in clinical practice. However, our previous study first found that cervical cancer has a broad range of α/β values across in vitro studies that follow a right‐skewed log‐normal distribution. If patient populations follow such a distribution, it may have potential impact on radiation therapy for cervical cancer.

**Purpose:**

To investigate the impact of variation in the α/β of cervical cancer on the expected EQD_2_ associated with clinical outcome for cervix cancer patients treated with radiation therapy and how that variance could influence the determination of alternate fractionation schemes.

**Methods:**

A right‐skewed log‐normal distribution of experimentally derived α/β values was applied to a reference tumor control probability (TCP) curve generated from cervical cancer patients treated with radiation, and a population of patients following that distribution were simulated using Monte Carlo sampling. An alternate equation for equivalent dose in 2 Gy fractions (EQD_2_) was derived that considered variance in α/β and was used to generate new values and associated TCP curves that could be plotted on a common EQD_2_ axis. Convolution curves of TCP and normal tissue complication probability (NTCP) were generated to determine the potential shift in optimal dose and probability of risk‐free local control (RFLC). Theoretical treatment failure rates were generated to evaluate changes in rates of treatment outcomes.

**Results:**

Variation in α/β obtained from published experimental results produced potential losses in TCP of up to 24% in the range of clinical interest. RFLC curves predicted an optimal treatment dose of 95 Gy EQD_2_ when applying our most probable α/β of 4.25 Gy, 10 Gy higher than that predicted by the reference curve. The α/β distribution saw a decrease in RFLC of 17%. To achieve a TCP of 90%, possible HDR fractionation schemes ranged from 56 Gy in 14 fractions to 32 Gy in 2 fractions, with the associated increase in normal tissue dose ranging from 11 to 16 Gy EQD_2_.

**Conclusion:**

The distribution of cervical cancer α/β values derived from experimental results produced significant changes in tumor control when applied to a reference TCP curve. TCP decreased with both the average and most probable α/β values. It is suggested that variance and heterogeneity in α/β be more explicitly incorporated to account for those patients that do deviate from the assumed constant value, especially in the case of evaluating different radiation schemes.

## 1 INTRODUCTION

Standard clinical treatment for locally advanced cervical cancer involves concurrent chemotherapy and external beam radiotherapy (EBRT) followed by an additional treatment of high dose rate (HDR) brachytherapy.^1^ Understanding how cervical cancer will respond to each radiation modality is important in developing radiation plans that can achieve maximal control of the cancer while minimizing complications to surrounding normal tissue.

The linear‐quadratic (LQ) model of cell survival, its parameters α and β, and ratio α a/β, are standardly used for comparing doses and also fractionation regimes and predicting tissue responses.^2^

It is common clinical practice to assign an α/β of 10 Gy for cancers including cervical cancer.^3^ However, our previous research has found that the α/β of cervical cancer has a large spread of values, ranging from 1.06 Gy to 34.3 Gy with a most probable value of 4.25 Gy, based on the analysis of 98 published α/β values from 31 cell in vitro studies.^4^ In this paper we continued to examine the impact of the variation in α/β on tumor control probability (TCP) for cervical cancer patients, which has years of well‐established clinical relevancy.

Although heterogeneity in α/β for TCP has been studied,^5^, ^6^, ^7^, ^8^ many of these studies have analyzed the impact on prostate cancer,^5^, ^7^ with none so far looking specifically at cervical cancer. In addition, no studies so far have analyzed the impact of heterogeneity and uncertainty when TCP is a function of BED or EQD_2_, which themselves are dependent on α/β. The traditional BED and EQD_2_ equations assume a constant α/β when adding or comparing values.^9^ Values computed with one α/β cannot be directly compared or combined with another. When incorporating variation or uncertainty in α/β, this can create misleading estimates of TCP if it is a function of BED or EQD_2_.

Cervical cancer is commonly treated with a combination of EBRT and brachytherapy, which requires the combination of EQD_2_ values to gauge their relative contributions to the expected tumor control. This means that an incorrect assumption of α/β can lead to under or over‐estimation of expected tumor control, even if the dose response curve itself does not have α/β in its parameters. Prescriptions and adaptations to treatment plans are calculated and assessed in terms of EQD_2_, and it is important to have the correct level of expected tumor control associated with various plans.

In this study we demonstrated the impact of variation in α/β on TCP based on EQD_2_, especially the impact of the deviation of α/β from the assumed 10 Gy on clinical practice in radiation therapy (combined EBRT and HDR brachytherapy) for cervical cancer.

## 2 METHODS AND MATERIALS

### 2.1 EQD_2_ as a function of α, β, and α/β

The LQ model is standardly used for comparing radiation fractionation schemes. In the treatment of cervical cancer it has common utility in combining EBRT and HDR doses, with the HDR portion of treatment being delivered as a boost to a sub‐volume of the EBRT target.

The LQ model allows clinicians to determine the cumulative radiobiological dose of different radiation schemes. This is known as the biological effective dose (BED) and in its current accepted form it is defined by the equation

(1) $$BED=D\left(1+\frac{d}{\alpha /\beta }\right)-K\left(T-T_{k}\right)$$

where *D* is the total dose of the treatment plan, *d* is the dose per fraction, and *T* is the total treatment time. *K* and *T_k_* are factors to account for tumor repopulation during treatment, with *K* being the BED needed to offset one day's worth of regrowth. *T_k_* is the kickoff time, the point after the beginning of treatment when the effects of repopulation become significant. If T < T_k_ then the repopulation effect is assumed to be zero. In most clinics, the dose from non‐standard fractionation schemes is converted into a biological equivalent dose in 2 Gy fractions (EQD_2_) and is given by

(2) $$EQD_{2}=\frac{BED+K\left(T-T_{k}\right)}{\left(1+\frac{2\;Gy}{\alpha /\beta }\right)}$$

where *EQD_2_* is the total dose of a 2 Gy/fraction plan that would achieve the same biological effectiveness as the treatment plan and *T* is the total treatment time of that equivalent 2 Gy/fraction treatment, assuming no gaps in treatment.

For cervical cancer, it is common practice to assign an α/β of 10 Gy. However, our previous research suggested a wide distribution of reported α/β values which peaked at 4.25 Gy^4^. One of the utilities of BED and EQD_2_ is in assessing how a tissue may respond to different radiation fractionations and evaluating different schemes that would have equal effectiveness. Take for example, a hypothetical 50 Gy plan at 2 Gy/fraction and we wished to find an equivalent around 7 Gy/fraction hypofractionated plan. Assuming an α/β of 10 Gy would yield a total required dose (for simplicity, repopulation is not accounted for) of about 5×7 Gy = 35 Gy. Yet changing α/β to 4.25 Gy would reduce that to approximately 4×7 Gy = 28 Gy, and an even lower α/β of 3 Gy down to a total dose of 4×6.5 Gy = 26 Gy. α/β has a significant effect when trying to determine the total dose for different fractionations. This usage of EQD_2_ and BED assumes a constant α/β^9^.

**FIGURE 1:**
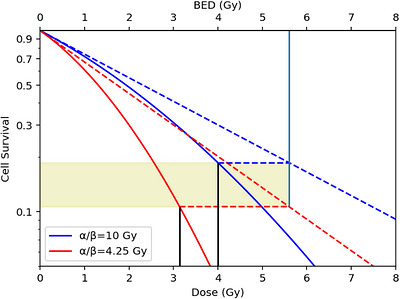
Cell survival curves for α/β = 10 Gy and α/β = 4.25 Gy. The angled dashed lines represent the linear portion of the linear quadratic survival curve. The horizontal dashed lines represent the difference between the physical acute dose and its associated BED. The shaded region highlights different levels of cell survival corresponding to the same BED of 5.6 Gy, pointed out by the vertical blue line. The two vertical black lines point to two different physical acute doses (3.2 and 4 Gy) and correspond to different points on each survival curve that predict the same BED. Note: The values on the axes are not equivalent to each other and represent different concepts.

Figure 1 demonstrates this concept with two hypothetical cell survival curves defined by two different α/β values. We can see how for a given BED value a change in α/β would significantly change the levels of cell killing and thus different acute physical doses. Therefore, variation and heterogeneity in α/β can lead to different levels of cell survival for a given BED and therefore lead to different EQD2 values and different physical doses. This makes plotting TCP against EQD2 difficult, as each α/β requires its own axis to be plotted against. In this paper we will derive a way to plot variations in α/β on a common axis.

Assuming two irradiations have equal biological effectiveness, we shall derive an equation for EQD_2_ that results from variation in α/β. Assume two levels of cell survival, *S* and *S’*, that correspond to the same BED, but are associated with different survival curves. Using equation 1 this relationship becomes

(3) $$\frac{-\ln\left(S\right)}{\alpha }=\frac{-\ln\left(S^{\prime }\right)}{\alpha^{\prime }}=BED$$

where *α* and *α’* represent the α parameters associated with each survival curve. If we wished to plot against BED, we would need to know the α value and corresponding survival level for each curve. Choosing to plot against EQD_2_ simplifies this. Dividing each side by *α’/β’*, we have

(4) $$EQ{D_{2}}^{\prime }=\frac{BED+K\left(T-T_{k}\right)}{\left(1+\frac{2\;Gy}{\alpha^{\prime }/\beta^{\prime }}\right)}\;$$

We now have a new EQD_2_ equation that accounts for variation in α/β, where *α/β* is the original value, in this case 10 Gy, and *α’/β’* is the new varied value. This new equation for EQD_2_ was used for all further calculations in our study. As *T* is dependent on EQD_2_’ we applied the methodology derived by Dale et al to remove that interdependency, assuming 2 Gy per day delivered 5 days per week. The details of the methodology are laid out in their paper.^10^ Based on the results of Gasinska et al^11^ and Tornero‐López et al,^12^ a value of 0.6 Gy/day was chosen for *K*. Huang et al estimated a kickoff time of 19 days^13^ for cervical cancer and this value was used in our calculations.

### 2.2 Reference dose response curve

A published TCP curve derived from a cervical cancer study performed by Dimopoulos et al^14^ was used as the refence TCP curve for this paper. Their study had three TCP curves derived from three‐year local control rates of the HRCTV D_90_ for cervix cancer patients, with all patients receiving a radiation prescription of 45–50.4 Gy external beam radiation therapy (EBRT) at 1.8 Gy/fraction delivered in a 3D 4‐field box, and 4 × 7 Gy high dose‐rate (HDR) brachytherapy. Their TCP curves were generated as a function of EQD_2_, assuming an α/β of 10 Gy. Probit regression techniques were used to derive their dose response curves.

(5) $$R=0.5+0.5\mathrm{erf}\left(\frac{t}{\sqrt{2}}\right),\;t=\frac{EQD_{2}-\;D_{50}}{{\left(\gamma \sqrt{2\pi }\right)}^{-1}\times D_{50}}\;$$

where *D_50_* is EQD_2_ that corresponds to 50% TCP and *γ* is a parameter that defines the steepness of the TCP curve. The parameters of the curves themselves make no estimates of α/β, meaning this study only examines the effect of α/β variation on the assumed α/β for EQD_2_. The curve chosen for this study (77 patients, D_50_ = 61 Gy, γ = 1.1) serves as a representation of EBRT plus brachytherapy treatments for cervical cancer.

### 2.3 Convolution and Monte Carlo simulation

We used Monte Carlo sampling methods to apply our α/β distribution to the reference TCP curve to generate a corresponding distribution of alternate TCP curves. We simulated hypothetical patient sub‐populations that deviated from the assumed α/β of 10 Gy, following the wide distribution of values estimated in our previous study.^4^ A custom‐made Monte Carlo simulation algorithm was developed on Python 3.10 and used to generate our altered TCP curves.

It was assumed that physical doses and recorded patient outcomes in the study by Dimopoulos et al^14^ were correct, and that variance came in the form of the radiobiological parameters and associated EQD_2_ values, which assumed α/β = 10 Gy. The reference TCP curve was convolved with the α/β distributions simulated over the range of clinical interest (RoCI), which represents a total cervical cancer radiotherapy dose of 70–85 Gy_10_ EQD_2_. To simulate a population of patients who's α/β followed our distribution, Monte Carlo sampling was applied. Moving across the RoCI in 1 Gy increments, clinical data was simulated at each treatment dose by randomly sampling from our distribution of α/β values 10,000 times and generating EQD_2_ values. Each sample would represent a hypothetical patient and their associated α/β value.

These methods are similar those employed by Nesvacil et al to simulate dosimetric uncertainty.^15^ Moving across the RoCI in 1 Gy increments, our α/β distribution was sampled 10,000 times, with each sample representing a hypothetical patient and their associated α/β value. For each sample a random number between 0 and 1 was chosen and evaluated against the probability of tumor control from the reference curve at the EQD_2_ estimated using equation 4. If the chosen number was lower than reference probability, the patient was assigned a treatment response, and if it was greater, a treatment non‐response. This was used to generate a binary clinical outcome for each treatment which was then fed into a logistic regression algorithm that used maximum‐likelihood estimation to generate new TCP curves. TCP curves were also generated for the most probable α/β value of 4.25 Gy we derived for cervical cancer. As a singular α/β was assumed in this case Monte Carlo sampling did not need to be applied.

### 2.4 Analysis of shift in optimal dose

While TCP curves quantify the probability of tumor control for a given dose, normal tissue complication probability (NTCP) curves represent the probability of normal tissues presenting a certain grade of radiation‐induced toxicity. The area between these curves represents a “therapeutic window” for physicians that aids in determining at what prescribed doses they can maximize tumor control while minimizing risk of complications to normal tissue.

Proposed by Schultheiss et al,^16^ and used by Boyer et al,^17^ the convolution between the TCP and NTCP of a treatment quantifies the probability of risk‐free local control (RFLC) and can be used to find where the therapeutic window is largest. They define the function as:

(6) $$RFLC\left(D\right)=TCP\left(D\right)\left(1-NTCP\left(D\right)\right)$$

where *TCP(D)* and *NTCP(D)* are response curves of the tumor and normal tissue, respectively, and *RFLC(D)* is the convolution of the two curves. As *NTCP(D)* gives us the probability of normal tissue toxicity, subtracting it from one gives us the probability of non‐complications for a given dose. Therefore, multiplying this value by *TCP(D)* will give us the probability of RFLC.

By plotting this function over a range of dose values we can see where it peaks and determine what the optimal dose would be. The convolution of the dose response curves was compared to determine the potential shift in optimal dose and what the change in RFLC would be. For the purpose of calculating NTCP, a reference curve derived by Georg et al^18^ was used, which analyzed grade ≥ 2 complications for the rectum and bladder in cervical brachytherapy treatments. In their study, the α/β of normal tissue was assumed to be 3 Gy, as is conventional in clinics and supported by previous research.^19^, ^20^, ^21^ In addition, convolution curves for alternate α/β values were generated that would achieve the same level of RFLC as the reference by adjusting expected dose to normal tissue.

### 2.5 Determination of treatment failure rate

To simulate hypothetical treatment failure rates, our event simulator, referenced in the previous section, recorded all simulated treatment outcomes and summed the total number of successes and total number of failures. This was done to simulate differences in expected treatment failure for different parts of the distribution. The study from which we referenced our cervical cancer dose response curve defined treatment failure as any local tumor recurrence or failure to remove local disease.^14^

The 3‐year treatment failure rate was then represented as the percentage of all simulated patients that had non‐response. Calculation of failure rates was repeated 30 times to assess the range of uncertainty in values and to satisfy the central limit theorem.

### 2.6 Determination and evaluation of alternative fractionation schemes

Although equation 4 tells the EQD_2_ associated with variation in α/β, it does not tell us the physical dose associated with that variation. As EQD_2_ is dependent on total dose and dose per fraction, there are multiple solutions for a given value. In this study we calculate a range of possible total and fractional doses that satisfy the alternate EQD_2_ values we derived.

In the study performed by Dimopoulos et al,^14^ their patients received a maximum prescription dose of 50.4 Gy EBRT in 28 fractions followed by 4 × 7 Gy brachytherapy, corresponding to a TCP of 90% when converted to EQD_2_, assuming α/β = 10 Gy. Change in α/β would change EQD_2_ and the dose–response curve, and therefore the fractionation schemes that would achieve that level of tumor control. Referencing the results of the EMBRACE‐I multicenter study,^22^ we defined a TCP of 90% as our minimum acceptable level of tumor control, and a range of fractionation schemes were determined that would meet that criteria for our alternate α/β values.

We also analyzed the relative impact such changes in fractionation would have on normal tissue, assuming an α/β of 3 Gy. Due to the greater possibly for alternative fractionation in the brachytherapy portion of treatment, and the generally large overlap of targets and organs at risk in EBRT, it was decided to analyze primarily variation in HDR regimens, evaluating fractional doses ranging from 4 to 16 Gy, and total doses of 16 to 50 Gy.

### 2.7 Comparison of cell histologies

Our previous research into the α/β of cervical cancer suggested a possible difference in radiosensitivity between cervical adenocarcinomas and squamous cell carcinomas, with the former having a slightly lower α/β. In addition to analyzing the potential impact our overall α/β distribution would have on clinical outcome and tumor control, individual cell histologies were also analyzed and compared, with all previous methods being applied to the α/β distributions of each histology.

## 3 RESULTS

### 3.1 Effect of α/β variation on TCP

**FIGURE 2:**
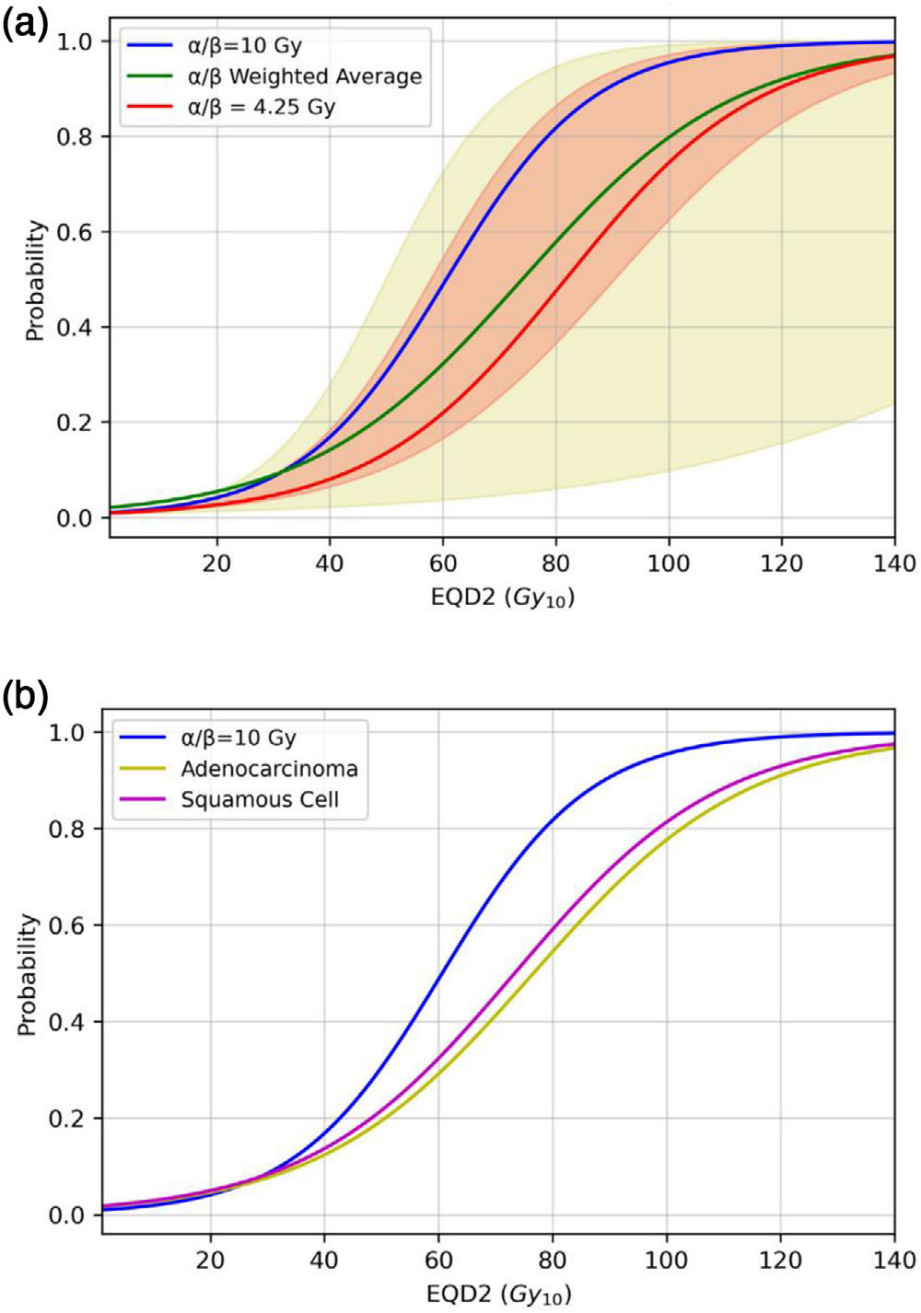
(a) TCP curves generated from the weighted average of sampling from our α/β distribution and from the most probable value of 4.25 Gy. The red shaded region represents the 68% confidence interval of our distribution, and the yellow shaded region represents the maximum extent of TCP variation (in yellow) generated by the published α/β values. Left edge of TCP range represents lowest α/β of 1.06 Gy and right edge represents highest value of 34.29 Gy. (b) TCP curves generated from the distributions of the squamous cell and adenocarcinoma histologies.

As can be seen in Figure 2(a), variation in α/β led to an overall decrease in TCP, although the extent of the distribution allows for increases in TCP for α/β values higher than 10 Gy. Figure 2(b) compares adenocarcinomas and squamous cell carcinomas, with adenocarcinomas having a slightly lower TCP, which agrees with the slightly lower α/β we witnessed in our earlier research. The weighted average of our α/β distribution produced a maximum loss in TCP of about 24%, with the most probable α/β of 4.25 Gy producing a decrease in TCP of about 31%. Adenocarcinomas had a slightly larger decrease than squamous cells, at about 25% versus 21%.

### 3.2 Analysis of shift in optimal dose

**FIGURE 3:**
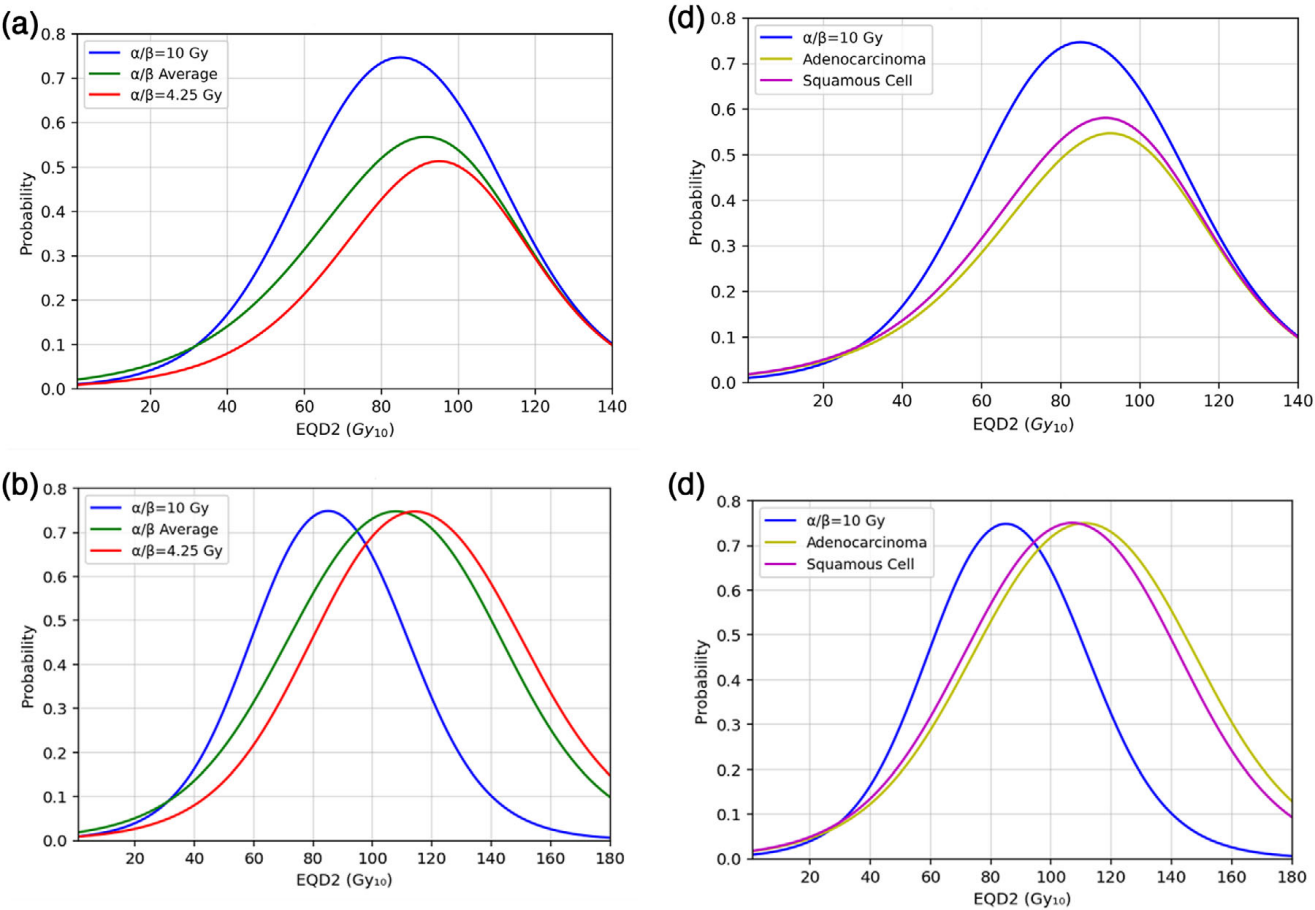
(a) Convolution curves demonstrating the probability of RFLC for our reference TCP of α/β = 10 Gy, the weighted average of applying our α/β distribution, and our most probable α/β of 4.25 Gy, applied to the reference prescription of 45–50.4 Gy EBT plus 4 × 7 Gy HDR brachytherapy. (b) Convolution curves generated for the α/β distribution and α/β of 4.25 Gy that achieve the same level of maximum RFLC as the reference curve (74%). (c) Convolution curves from the reference prescription and (d) those with equal RFLC as the reference curve for the adenocarcinoma and squamous cell histologies.

Assuming a normal tissue α/β of 3 Gy, our reference curve, published by Dimopoulos et al,^10^ had an optimal EQD_2_ of 85 Gy, at an associated RFLC probability of 74%. Table 1 summarizes the optimal EQD_2_ and associated RFLC probability for each curve. Our most probable α/β gave the largest deviation in optimal EQD_2_ from our reference, with a value of 95 Gy and an associated probability of RFLC of 51%. It also had the largest shift in optimal dose when attempting to achieve the reference 74% RFLC, at 108 Gy EQD_2_. Figure 3 highlights the changes in our reference convolution curve, with figure 3(a),(b) representing our total distribution and figure 3(c),(d) representing the histology‐based distributions. In all cases there was an increase in optimal EQD_2_ with an associated loss in RFLC. Squamous cells and adenocarcinomas were almost identical in the curves they generated, with adenocarcinomas having a 3% lower peak RFLC than squamous cells. In addition, convolution curves were generated for all cases that achieved the same maximum RFLC as the reference curve at 74%. This corresponded to much larger EQD_2_ values, being as large as 108 and 114 Gy for the weighted average and α/β = 4.25 Gy respectively.

**TABLE 1:** Comparisons of optimal doses in EQD_2_ and corresponding probability of RFLC for each of our sets of generated clinical data. The 2^nd^ row corresponds to generated convolution curves that achieve the same level of maximum RFLC as the reference curve.

α/β = 10 Gy	α/β Average	α/β = 4.25 Gy	Adenocarcinoma	Squamous cell
85 Gy, 74%	92 Gy, 57%	95 Gy, 51%	93 Gy, 55%	91 Gy, 58%
85 Gy, 74%	108 Gy, 74%	114, 74%	110 Gy, 74%	106 Gy, 74%

### 3.3 Treatment failure rate

Our most probable α/β saw the largest increase in treatment failure rates, with an increase of 29.9% over the reference rate of 16.3%. Table 2 summarizes predicted treatment failure rates and associated 68% confidence intervals when applicable for each TCP curve. When comparing histologies, squamous cell carcinomas had the lower treatment failure rate at around 36%, versus squamous cell carcinoma, which had a rate of about 40%, though squamous cells had a wider confidence interval.

**TABLE 2:** Treatment failure rates within the range of delivered treatment (45–50.4 Gy x 25 fx EBRT plus 4x7 Gy HDR brachytherapy) and their corresponding standard deviations^*^ for each of our three sets of generated clinical data.

α/β = 10 Gy	α/β Distribution	α/β = 4.25 Gy	Adenocarcinoma	Squamous cell
16.3%	37.4 + 14.2%, 37.4 − 16.6%	46.2%	40.3 + 13.7%, 40.3 − 15.6%	36.1 + 14.3%, 36.1 − 16.4%

*Note*: ^*^ Reference value and most probable α/β did not have associated confidence intervals, as a singular value was assumed through all calculations.

**FIGURE 4:**
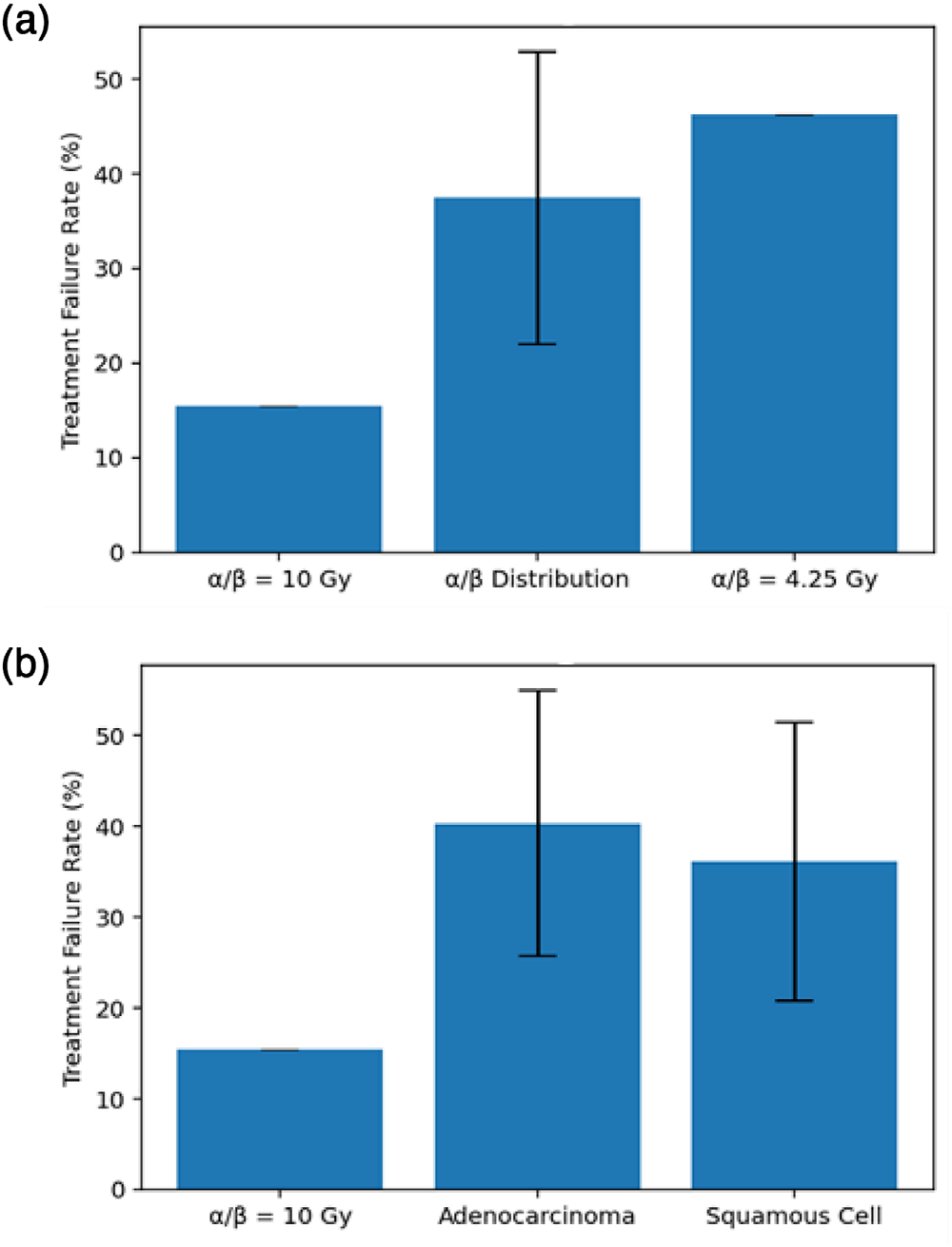
(a) Predicted treatment failure rates for our α/β distribution and most probable α/β of 4.25. (b) Predicted treatment failure rates of adenocarcinomas and squamous cell carcinomas. Black bars represent the 68% confidence interval for each distribution. The most probable α/β did not have associated confidence intervals as a singular value was assumed throughout all calculations.

Figure 4 shows just how much the overall treatment failure rate changed for each set of generated clinical data. The error bars at the top of the bar plots represent the 68% confidence intervals for each distribution, with the most probable α/β not having one due to assuming a singular α/β value through all calculations.

### 3.4 Determination and evaluation of alternative fractionation schemes

**FIGURE 5:**
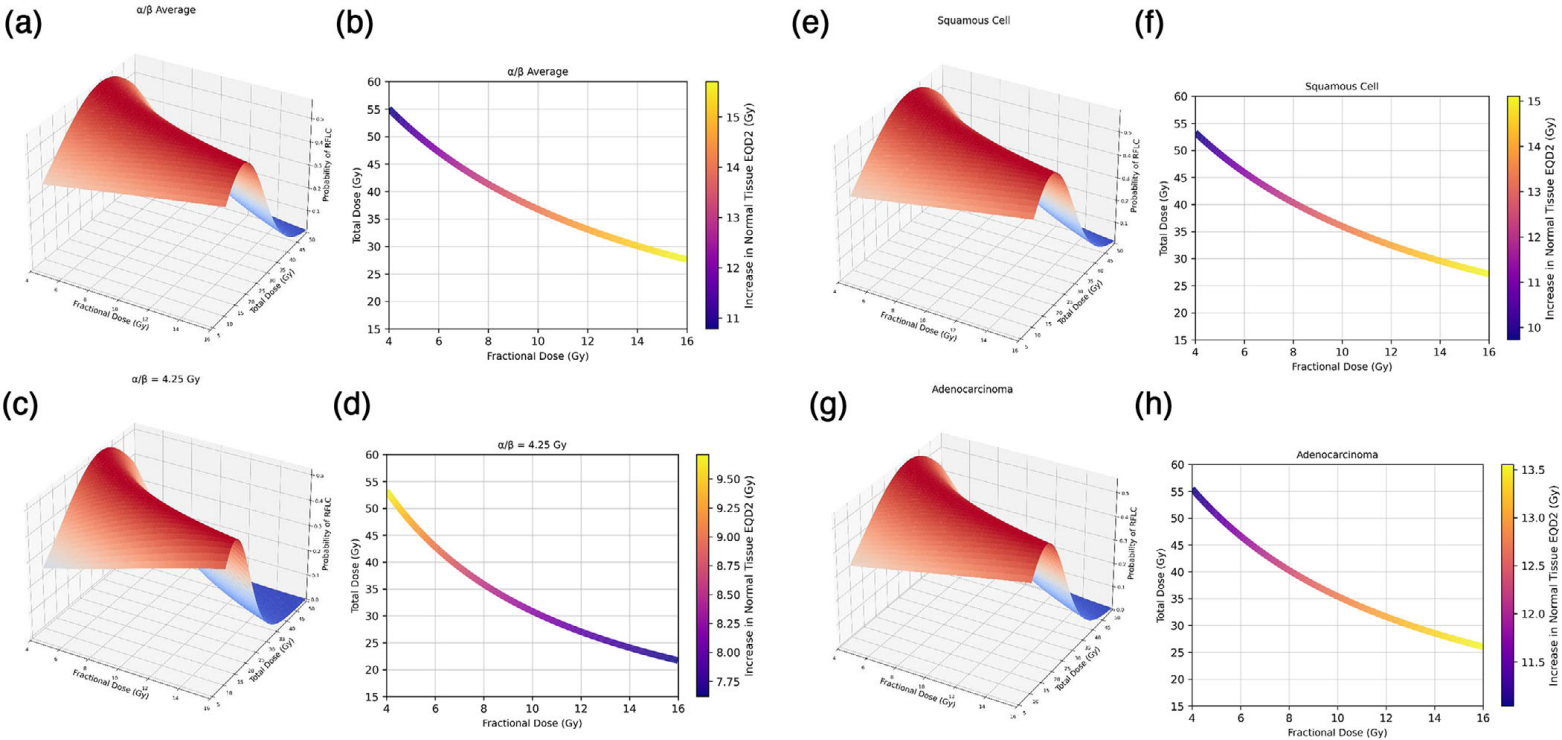
Two‐dimensional plots of convolution curves and the probability of RFLC as a function of fractional dose and dose for the HDR brachytherapy portion of treatment; plotted for (a) weighted average of α/β distribution, (c) α/β = 4.25 Gy, and for weighted average of (e) squamous cell and (g) adenocarcinoma histologies. In addition, curves were generated that showed the fractionation schemes that achieved 90% TCP, with associated heat map demonstrating the increase in normal tissue EQD_2_ from the reference TCP curve; plotted for (b) weighted average of α/β distribution, (d) α/β = 4.25 Gy, and for weighted average of (f) squamous cell and (h) adenocarcinoma histologies.

As can be seen in Figure 5, when plotting possible fractionation schemes as a function of RFLC, the most probable α/β resulted in a slightly wider range of optimal total doses for HDR than the overall average. Similar behavior was seen when comparing cell histologies, with adenocarcinomas having a slightly wider range. When analyzing fractionation schemes that would achieve a 90% level of tumor control, our most probable α/β predicted the maximum total dose of about 52 Gy in 13 fractions and the lowest total dose of about 22 Gy in 2 fractions. All fractionation schemes for every group saw some level of increase in normal tissue EQD_2_, with the most hypofractionated schemes producing the largest increase, at 16 Gy EQD_2_ for about 16 Gy in 2 fractions in the case of the average. To limit dose to the OARs, we recommend fractional doses up to 10 Gy.

## 4 DISCUSSION

Our previous paper demonstrated the potential impact of our derived α/β distributions on the dosimetry of the treatment of cervical cancer,^4^ suggesting that it would lead to potentially significant changes in clinical outcome. In this work we demonstrated the potential clinical impact of large heterogeneity in α/β when applied to EQD_2_, producing results that we deem to not only be statistically significant, but clinically significant as well based on the results of the TCP and convolution curves. Using Monte Carlo methods to simulate outcome data and generate alternate TCP curves, this study is the first of its kind to analyze the effect of α/β heterogeneity on clinical cervical cancer data.

As the standard BED and EQD_2_ equations assume a constant α/β, variability in the ratio forced us to redefine EQD_2_ as a function of variance in α/β. The impact on TCP was significant, with our overall distribution of α/β values producing a decrease in the range of clinical interest of up to 24%. The most probable α/β for cervical cancer derived from our previous study saw the largest deviation from reference values, with a decrease in TCP of up to 31%, and an increase in treatment failure rates of over 20%.

The reference convolution curve, with α/β = 10 Gy, predicted an optimal total dose of about 85 Gy EQD_2_ with an RFLC of 74%, which is in line with the 80–90 Gy EQD_2_ that many clinics prescribe to for cervical cancer.^23^ Our most probable α/β value of 4.25 Gy suggested a slightly higher optimal dose of approximately 95 Gy EQD_2_ with an RFLC of 51%, resulting a loss of 23% in RFLC. The loss in RFLC suggests that applying the prescription dose used in this study may correspond to unexpected and significant changes in clinical outcome for those subpopulations of patients whose α/β deviate from the assumed value. In addition, generating convolution curves that could achieve the same level of RFLC (74%) as the reference curve predicted much higher optimal doses, with the α/β of 4.25 Gy predicting and EQD_2_ of 114 Gy (Table 1), corresponding to at least two extra fractions in the HDR portion of the reference prescription or up to 12 extra fractions in the external beam portion. This suggests a dose escalation for low α/β tumors may be needed. But dose escalation should consider the OAR's dose limits.

To achieve these higher levels of RFLC of course necessitates the reduction of normal tissue dose, suggesting a potential benefit from adopting more conformal treatment plans for those clinics that still used older, less conformal treatment methodologies. Our previous research also suggested a wide distribution of α/β values, and this is just one example of how, in a heterogeneous population, deviations from the assumed α/β can have significant implications for those cases that do deviate from the norm.

To maintain a TCP of at least 90%, we calculated possible fractionation schemes ranging from 56 Gy in 14 fractions for all groups, down to at least 32 Gy in 2 fractions for α/β = 4.25 Gy. Although the smaller dose per fraction plans generally had the lowest increase in normal tissue dose, they necessitated the largest amount of total dose, extending the possible treatment time. At the reference fractionation of 7 Gy per fraction, the overall α/β distribution necessitated a total HDR dose of at least 44 Gy, 16 Gy higher than the reference dose, and requiring a total of 7 fractions. As many HDR treatments are administered 2 to 3 times a week, this could extend the treatment by at least a week. The American Brachytherapy Society recommends that the entire cervical cancer radiation treatment be completed within 8 weeks.^24^ If we wanted to reduce the total treatment by maintaining 4 treatment fractions, that would require 10 Gy per fraction, a 43% increase in fractional dose. However, the OAR dose limits should be considered while applying the higher fractional doses. Especially, in the cases where OARs are adjacent to or even overlap with clinical targets, it may not be feasible to prescribe more extreme fractional doses to exceed the OAR dose limits. In this situation, the assessment of the tradeoff between effectively controlling the cancer and limiting damage to OARs would be performed to help make a clinical decision. The results above demonstrate not necessarily a drastic shift in treatment for all cervical cancer patients, but shows the impact to those patients that deviate from the best‐fitted TCP curve, especially if one were to explore alternative fractionation schemes.

It is understood that a dose response curve fitted to heterogenous population will not necessarily be predictive of all patients in that population, but this paper suggests that for those patients that do deviate in terms of their α/β, the impact could be significant and steps should be taken to better address this spread of expected patient outcomes. In addition, with radiation therapy for cervical cancer being a multi‐modal treatment, expression of dose in EQD_2_ is necessary to measure the relative impact of each treatment and make appropriate shifts to treatment when necessary. This of course is dependent on α/β. With the potential for a highly heterogenous distribution of values, ignoring variance in that parameter and applying a blanket value may be sub‐optimal for those patients that deviate from the norm.

The results of our previous study^4^ suggested an average α/β for adenocarcinomas that was slightly lower than squamous cell carcinomas and although the results here support this pattern, the magnitude of the differences were very minor, with TCP curves of the two for example having a coefficient of determination (R^2^) of 0.97. Similarly, extremely close values were seen in all other results. Clinical trials have demonstrated clear differences in local control rates for squamous cells and adenocarcinomas,^25^ with the latter having poorer outcomes. The results of this study may suggest that external factors beyond inherent cellular radiosensitivity may be the influencing factor in the differences seen in the clinic.

It must also be mentioned that in the study by Dimopoulos et al,^14^ only 11% of their patients had adenocarcinoma, and it is likely that their derived dose response curve was primarily influenced by the much larger squamous cell patient group. This may also play a role in the results we see when comparing histologies and is unfortunately a common trend with many such studies, due to the relative rarity of adenocarcinomas in cervical cancers. It is a direction of future research for the authors of this paper to collect individual patient data and categorize them based on cancer cell histology.

The reference dose response curve used in this study^14^ is a heterogeneous TCP model designed to better fit the shallower dose responses seen in patient data. It had two fitting parameters, D_50_ and γ, which represent the dose at 50% tumor control and the steepness of the curve, respectively. The parameters themselves make no estimates of α/β, but the TCP curve was a function of radiobiological dose, rather than physical dose, that assumed α/β = 10 Gy. As mentioned earlier, prescriptions and plan adaptations in cervical cancer treatment are often given in EQD_2_ to assess the relative impact of the external beam and HDR portions of the treatment. The results here show that even if the parameters of the dose response curve itself makes no estimates of radiobiological parameters, inputing an inaccurate EQD_2_ estimate can have a significant effect on what doses would achieve expected levels of tumor control.

## 5 CONCLUSION

The results of this study demonstrated that the distribution of α/β values from in vivo experimental studies may have a potentially significant clinical impact. We showed that variation in α/β can have different effects on EQD_2_ and dose response curves when they are a function of EQD_2_, as is often the case with cervical cancer treatment. We also demonstrated that variation in experimental α/β values produced significant changes in tumor control when applied to a reference TCP curve based on the α/β of 10 Gy for cervical cancer. Both the average and most probable values of the α/β distribution significantly reduced the TCP values. It is suggested that variance and heterogeneity in α/β be more explicitly incorporated to account for those patients that do deviate from the assumed constant value, especially in the case of evaluating different radiation schemes.

## AUTHOR CONTRIBUTIONS

***Conceptualization, formal analysis, investigation, methodology***: Cameron Thayer‐Freeman, Wei Luo; ***Software***: Cameron Thayer‐Freeman, Brien Washington; ***Original draft***: Cameron Thayer‐Freeman; ***Review & editing***: Cameron Thayer‐Freeman, Brien Washington, Dennis Cheek, Wei Luo; ***Supervision***: Wei Luo.

## CONFLICT OF INTEREST STATEMENT

The authors declare no conflicts of interest.
